# Prospective study of superior cluneal nerve disorder as a potential cause of low back pain and leg symptoms

**DOI:** 10.1186/s13018-014-0139-7

**Published:** 2014-12-31

**Authors:** Hiroshi Kuniya, Yoichi Aota, Takuya Kawai, Kan-ichiro Kaneko, Tomoyuki Konno, Tomoyuki Saito

**Affiliations:** Department of Orthopaedic Surgery, Yokohama City University Graduate School of Medicine, Fukuura 3-9, Kanazawa-ku, Yokohama City, Kanagawa 236-0004 Japan; Department of Spine and Spinal Cord Center, Yokohama Brain and Spine Center, Takigashira 1-1-2, Isogo-ku, Yokohama City, Kanagawa 235-0012 Japan

**Keywords:** Superior cluneal nerve, Entrapment neuropathy, Low back pain, Leg pain, Nerve block injection

## Abstract

**Background:**

Entrapment of the superior cluneal nerve (SCN) in an osteofibrous tunnel has been reported as a cause of low back pain (LBP). However, there are few reports on the prevalence of SCN disorder and there are several reports only on favorable outcomes of treatment of SCN disorder on LBP. The purposes of this prospective study were to investigate the prevalence of SCN disorder and to characterize clinical manifestations of this clinical entity.

**Methods:**

A total of 834 patients suffering from LBP and/or leg symptoms were enrolled in this study. Diagnostic criteria for suspected SCN disorder were that the maximally tender point was on the posterior iliac crest 70 mm from the midline and that palpation of the tender point reproduced the chief complaint. When patients met both criteria, a nerve block injection was performed. At the initial evaluation, LBP and leg symptoms were assessed by visual analog scale (VAS) score. At 15 min and 1 week after the injection, VAS pain levels were recorded. If insufficient pain decrease or recurrence of pain was observed, injections were repeated weekly up to three times. Surgery was done under microscopy. Operative findings of the SCN and outcomes were recorded.

**Results:**

Of the 834 patients, 113 (14%) met the criteria and were given nerve block injections. Of these, 54 (49%) had leg symptoms. Before injection, the mean VAS score was 68.6 ± 19.2 mm. At 1 week after injection, the mean VAS score significantly decreased to 45.2 ± 28.8 mm (*p* < 0.05). Ninety-six of the 113 patients (85%) experienced more than a 20 mm decrease of the VAS score following three injections and 77 patients (68%) experienced more than a 50% decrease in the VAS score. Surgery was performed in 19 patients who had intractable symptoms. Complete and almost complete relief of leg symptoms were obtained in five of these surgical patients.

**Conclusions:**

SCN disorder is not a rare clinical entity and should be considered as a cause of chronic LBP or leg pain. Approximately 50% of SCN disorder patients had leg symptoms.

**Electronic supplementary material:**

The online version of this article (doi:10.1186/s13018-014-0139-7) contains supplementary material, which is available to authorized users.

## Background

The superior cluneal nerve (SCN) is derived from the cutaneous branches of the dorsal rami of T11-L4 [1-3]. A relationship between the cluneal nerve and low back pain (LBP) was sporadically reported several decades ago. The first detailed description was made by Strong and Davila in 1957 [4], who attempted deafferentation of the SCN in 30 patients when that nerve was considered to cause LBP. Although the chief complaint was limited only to LBP in their series, 21 patients had referred pain in various areas of the leg.

Maigne [3,5] drew attention to the so-called “Maigne’s syndrome”, which involves facet syndromes at the thoraco-lumbar junction causing unilateral LBP. In this syndrome, the pain is not experienced at the junction but is referred lower to the dermatomes of corresponding cutaneous dorsal rami. Pressure on the iliac crest reproduces the pain; this point corresponds to the emergence of the SCN [3]. Maigne described multiple sources of pain around a joint, not just the facets [6]. Sato described that patients with vertebral fractures at the thoraco-lumbar junction often experience referred LBP around the iliac crest, following the course of the SCN [7]. This seems to be an example of “Maigne’s syndrome”.

Maigne also drew attention to the spontaneous entrapment of the SCN in an osteofibrous tunnel in the space surrounded by the iliac crest and thoraco-lumbar fascia in LBP [3]. Following these reports by Maigne et al., several researchers found that the medial branch of the SCN consistently passed through an osteofibrous tunnel and may have been spontaneously entrapped in the tunnel [2,3,8,9]. More recently, Kuniya et al. noted that 39% of the medial branches of the SCN passed through an osteofibrous tunnel and only 5% of these exhibited macroscopically apparent entrapment [10]. There have been two case series [11,12] and two case reports [13,14] in English language journals describing successful treatment for LBP by SCN release. Successful surgical outcomes were obtained, notably when severe entrapment was observed during surgery [11]. However, previous surgical reports are very few and limited to a small number of subjects with unilateral low back and/or buttock pain [11-14].

It is generally known that injury to the branches of the SCN, as a complication of bone graft harvest from the posterior iliac crest for spinal fusion, is a cause of chronic LBP [15-17]. Trescot described that cluneal neuralgia was more commonly the result of a spontaneous entrapment of the nerve rather than of a nerve injury during bone harvest. This clinical entity may be underdiagnosed and should be considered as a potential cause for chronic LBP or leg pain [18]. To date, there have been few reports assessing the prevalence of SCN disorder. Although there were several studies that described treatment by nerve block injection for SCN disorder, detailed outcomes evaluated using the visual analog scale (VAS; maximum score 100 mm) were not included in these reports [19-22].

The purposes of this study were fourfold: 1. to determine the prevalence of SCN disorder in spine clinic patients, 2. to determine the relationship between SCN disorder and fractures in the thoraco-lumbar junction, 3. to evaluate the outcomes of nerve block injections, and 4. to present surgical outcomes in patients who had intractable symptoms.

## Methods

This prospective study was conducted from 2009 to 2013. A total of 834 consecutive patients (394 females and 440 males, average age 64 years, age range 16–94 years) visiting the Department of Spine Surgery at our institution with the primary complaint of LBP and/or leg symptoms were registered for this study. Patients with prior bone harvest from the posterior iliac crest were excluded. X-rays of the lumbar spine and thoraco-lumbar junction were taken. When patients had leg symptoms, magnetic resonance imaging (MRI) of the lumbar spine was routinely performed to examine the spinal canal and foraminal pathology.

The diagnostic criteria for suspected SCN disorder were the following: 1) the maximal tender point was on the posterior iliac crest approximately 70 mm from the midline and 45 mm from the posterior superior iliac spine where the medial branch of the SCN runs through an osteofibrous tunnel consisting of the thoraco-lumbar fascia and the iliac crest and 2) palpation of the maximally tender point reproduced the chief complaint of LBP and/or leg symptoms. When patients met both criteria, a nerve block injection was performed.

At the initial evaluation, LBP and leg symptoms were assessed by VAS and the Roland-Morris Disability Questionnaire (RDQ). At 15 min and 1 week after the injection, VAS pain levels were recorded and the incidence rate of complications was also determined. If insufficient pain decrease or recurrence of pain was observed, injections were repeated weekly up to three times until sufficient pain relief was obtained.

Surgery was indicated only when temporal pain relief was obtained after SCN blocks and repetitive SCN blocks failed to produce sufficient pain relief.

Surgeries and this prospective study were approved by the Ethics Committee of Yokohama City University and Yokohama Brain and Spine Center. The subjects were informed that SCN release was previously performed exclusively for patients with buttock and/or LBP [11-14] and had never been applied for leg symptoms. The patients gave their informed consent to undergo surgical decompression.

### Surgical methods

**Figure 1:**
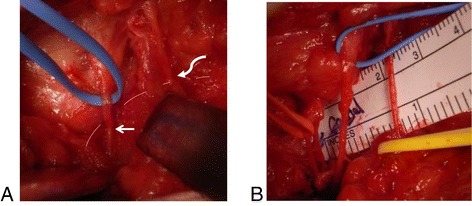
**Photos during surgical superior cluneal nerve (SCN) release in case 9.** Two branches of the SCN were identified within 5 cm above the iliac crest (dotted line) to be seen to emerge from beneath the lateral margin of the deep layer of fibro-thoraco-lumbar fascia. A blue tape has been used to lift and highlight a branch which remained compressed by the fascia (arrow). Curved arrow indicates another branch of SCN **(A)**. Underneath these branches, the two other anastomosing branches were identified and a total of four branches were released **(B)**.

All surgeries were performed by a senior author (YA). The skin over the operative site at tender points was marked using a marker pen just before surgery. SCN release was performed under general anesthesia, with the patient in the prone position, through a 7-cm oblique skin incision over the iliac crest. An operating microscope was used. While being careful not to injure nerve branches passing through the subcutaneous tissue, the superficial layer of the thoraco-lumbar fascia was opened. One or two branches of the SCN were identified within 5 cm above the iliac crest and were seen to emerge from the lateral margin of the deep layer of the thoraco-lumbar fascia (Figure 1A). SCN branches were traced in a caudal direction until they passed through the gluteal fascia into the subcutaneous tissue. Because a recent anatomical study by Kuniya et al. [10] indicated that one to three SCN branches may pass through the osteofibrous tunnel and that 2% of the specimens had severe constriction of the SCN within a bony groove on the iliac crest, we routinely explored all branches passing around the tender point by tracing each anastomosing branch until the rim of the iliac crest was explored (Figure 1B). The iliac crest beneath the nerve was removed using air drill, approximately fingertip in size, to obtain thorough decompression of the nerve. After decompression, the SCN branches were embedded within the wide bone groove just made and covered by sufficient subcutaneous fat to provide insulation and padding.

### Radiological analysis

Lateral X-rays were reviewed by an experienced spine surgeon (HK) to determine whether there was a fracture in any of the T10 to L5 visualized vertebrae. A fracture was defined as grade 1 by Genant semi-quantitative method (a reduction in vertebral height of 20%–25%) or more [23].

### Statistics

Comparisons between groups were made using Fisher’s exact test, Mann-Whitney’s *U* test, and the paired *t*-test. The level of significance was defined as 0.05. Analyses were performed using Statcel 2 statistical software, version 2 (OMS Inc., Japan).

## Results

**Figure 2:**
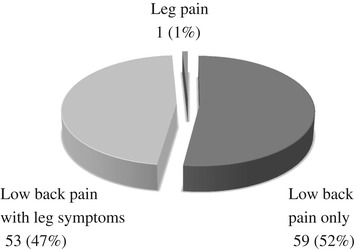
**Chief complaints of 113 subjects meeting the criteria for suspected superior cluneal nerve disorder.** Of the 113 subjects meeting both criteria, 59 (52%) have only low back pain (LBP), 53 (47%) have LBP with leg symptoms, and 1 (1%) has only leg pain.

Of the 834 registered patients, 122 had the maximally tender point on the posterior iliac crest and met the first criterion. Of these 122 patients, 113 (62 females and 51 males; average age 68 years; age range: 17–93 years) had their chief complaint reproduced by palpation of the maximally tender point, thus meeting both criteria. These subjects were diagnosed with suspected SCN disorder. Significantly, more women (*p* < 0.05) and older subjects (*p* < 0.05) were included in the suspected SCN disorder group than in the non-SCN disorder group (Table 1). The average disease duration of suspected SCN disorder subjects was 27.3 ± 56.5 months (range: 0.1–444.0 months) and there were no significant differences in the duration of the disease between the two groups. The average RDQ was 13.4 ± 5.6 (0–22) in the suspected SCN disorder group, a significantly higher level than in the non-SCN disorder group (*p* < 0.05). Of the 113 subjects, 59 (52%) had only LBP, 53 (47%) had LBP associated with leg symptoms, and 1 (1%) had only leg pain (Figure 2). Various postures and motions aggravated both LBP and leg symptoms in these 113 subjects (Table 2). The causes for aggravation of pain included walking (39 cases), rising from a seated position (33 cases), standing position (25 cases), forward bending (16 cases), backward bending (11 cases), supine position (7 cases), sitting position (6 cases), any motion (4 cases), putting weight on the affected side (2 cases each), lifting something heavy (2 cases each), twisting motion (2 cases each), getting up out of bed (2 cases each), decubitus position (2 cases), walking down the stairs (1 case), and prone position (1 case), while 5 patients stated no activity or posture aggravated their pain.

**Table 1:** **Demographic data at initial evaluation**

	**Suspected SCN disorder,** ***n*** **= 113**	**Non-SCN disorder,** ***n*** **= 721**	***p*** **value**
Female/male	62/51	332/389	<0.05
Average age (years, range)	68.2 ± 14.6 (17–93)	63.1 ± 16.4 (16–94)	<0.05
Average disease duration (months, range)	27.3 ± 56.5 (0.1–444.0)	28.5 ± 64.5 (0.1–542.7)	NS
Average RDQ (range)	13.4 ± 5.6 (0–22)	11.1 ± 6.5 (0–24)	<0.05
Average VAS (range)	68.6 ± 19.2 (25–100)	70.8 ± 25.2 (0–100)	NS
Presence of fractures at thoraco-lumbar or lumbar spine	26 (23%)	70 (10%)	<0.01

*SCN* superior cluneal nerve, *RDQ* Roland-Morris Disability Questionnaire, *VAS* visual analog scale, *NS* not significant.

**Table 2:** **Motions and postures aggravating symptoms in subjects with suspected SCN disorder (** ***n*** **= 113)**

**Motions and postures**	**Number**
Walking	39
Rising from seated position	33
Standing position	25
Forward bending	16
Backward bending	11
Supine position	7
Sitting position	6
Any motion	4
Putting weight on the affected side	2
Lifting something heavy	2
Twisting motion	2
Getting out of bed	2
Decubitus position	2
Walking down the stairs	1
Prone position	1
No activity aggravated symptoms	5

Multiple answers were allowed.

*SCN* superior cluneal nerve.

### Vertebral fractures

**Figure 3:**
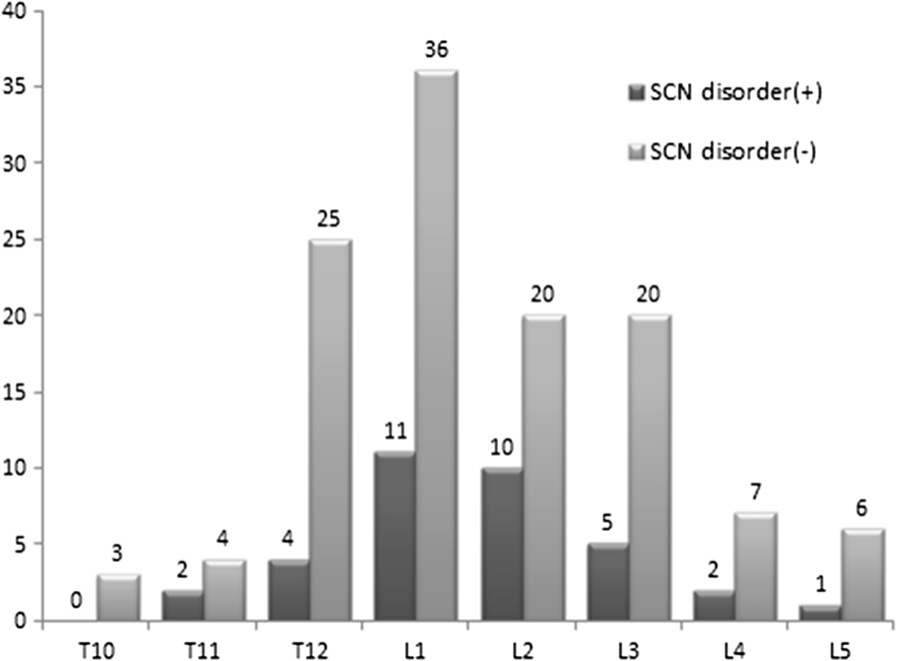
**Distribution of vertebral fractures.** Twenty-nine patients had multiple fractures.

In the 834 registered patients, compression fractures were found in a total of 156 vertebral bodies in 96 patients (38 males and 58 females) with a mean age of 74.8 ± 10.2 years (age range: 33–94), who experienced more than a 20 mm decrease of the VAS score following up to three times nerve block injections. The prevalence of SCN disorders was significantly higher in patients with vertebral fractures than in the remaining 738 patients without vertebral fractures (26/96 vs 87/738) (*p* < 0.01) (Table 3). The distribution in the level of vertebral fracture is shown in Figure 3. There was no significant difference in the distribution of the involved vertebrae.

**Table 3:** **Comparison between patients with and without vertebral fractures**

	**Patients with vertebral fractures (** ***n*** **= 96)**	**Patients without vertebral fractures (** ***n*** **= 738)**	***p*** **value**
SCN disorder	26 (27%)	87 (12%)	<0.05
Female/male	20/6	42/45	<0.05
Average age (years, range)	75.5 ± 5.0 (67–84)	66.1 ± 15.8 (17–93)	<0.05
Average disease duration (months, range)	24.4 ± 24.5 (0.5–89.5)	28.5 ± 64.7 (0.1–444.0)	<0.05
Average RDQ (range)	15.9 ± 5.1 (6–22)	9.5 ± 7.2 (0–22)	<0.05
Average VAS (range)	72.7 ± 17.2 (40–100)	67.3 ± 19.7 (25–100)	NS

*SCN* superior cluneal nerve, *RDQ* Roland-Morris Disability Questionnaire, *VAS* visual analog scale, *NS* not significant.

### Illustrative case report

**Figure 4:**
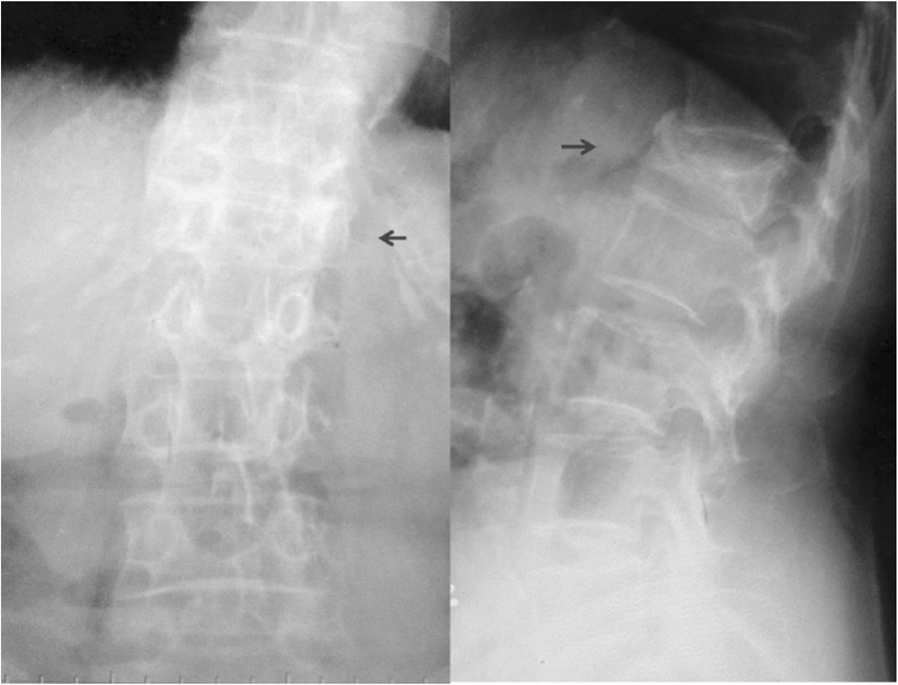
**X-ray in a 70-year-old SCN patient.** A collapsed vertebral fracture at L1 is shown (arrow).

A 70-year-old woman reported a 5-month history of disabling LBP on the left side. Following an initial diagnosis of L1 fracture (Figure 4), bracing was attempted but resulted in no pain relief. She had a history of Behcet’s disease and Sjögren syndrome. At percussion on the L1 spinous process, she had some discomfort. The maximally tender point was on the left iliac crest and pressure on the tender point clearly reproduced the pain. Complete pain relief was obtained by a single SCN block. The VAS score before injection was 80; VAS scores were 0 at 30 min and 1 week after the SCN block.

### Effect of SCN blocks

**Figure 5:**
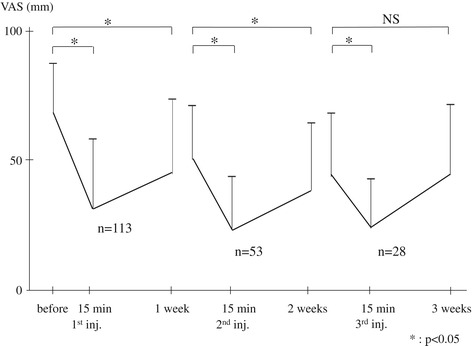
**Changes in VAS scores of 113 subjects suspected with superior cluneal nerve disorder.** The line graph shows the changes in VAS scores before, 15 min, and 1 week after nerve block injections of 113 patients meeting the both criteria for suspected superior cluneal nerve disorder. The mean VAS score is 68.6 ± 19.2 mm (25–100 mm) before injection. At 15 min after injection, the mean VAS score is 31.6 ± 27.0 mm (0–100 mm), a significant decrease (*p* < 0.05). At 1 week after injection, the VAS scores significantly decrease to 45.2 ± 28.8 mm (0–100 mm) (*p* < 0.05). If insufficient pain decrease or recurrence of pain was observed, injections were repeated every week up to three times, or until sufficient pain relief was obtained. Fifty-three (47%) patients required a second injection, and 28 (25%) patients required a third time injection.

The mean VAS score of the 113 patients meeting both criteria for suspected SCN disorder was 68.6 ± 19.2 mm (25–100 mm) before the SCN block, with no significant difference from that of the non-SCN group. Fifteen minutes after the initial injection, their mean VAS score was 31.6 ± 27.0 mm (0–100 mm), a significant decrease (*p* < 0.05). At 1 week after the initial injection, their mean VAS score had increased to 45.2 ± 28.8 mm (0–100 mm), still significantly (*p* < 0.05) decreased compared to their mean VAS score before injection (Figure 5).

Fifty-three (47%) of the suspected SCN disorder subjects required additional injections for remaining symptoms or recurrence of pain. Before the second injection, the mean VAS score of these subjects was 50.6 ± 20.9 mm (10–100 mm). Fifteen minutes after the second injection, their mean VAS score had significantly decreased to 23.4 ± 20.7 mm (0–70 mm) (*p* < 0.05). At 1 week after the second injection, their mean VAS score had increased to 38.6 ± 26.2 mm (0–90 mm), still significantly (*p* < 0.05) decreased compared to their VAS score before the second injection.

Of these subjects, 28 (25%) required a third injection. Before the third injection, the mean VAS score of these subjects was 43.9 ± 24.7 mm (10–90 mm). Fifteen minutes after the third injection, their mean VAS score had significantly decreased to 23.9 ± 19.3 mm (0–60 mm) (*p* < 0.05). At 1 week after the third injection, their mean VAS score was 44.6 ± 27.3 mm (0–80 mm), not significantly decreased from that before the third injection.

The number of subjects who obtained more than a 20 mm decrease of VAS score after the first injection was 91 (81%), after the second injection 93 (82%), and after the third injection 96 (85%). Similarly, the number of patients who obtained more than a 50% decrease of VAS score after the first injection was 70 (62%), after the second injection 76 (67%), and after the third injection 77 (68%).

A complication of the injection, erectile dysfunction (ED), occurred in two cases. One was a 41-year-old male who experienced temporary ED during pain reduction after every injection (see below in the sections of “Surgical case reports”). The other case was permanent ED, which occurred immediately after the first injection in a 65-year-old male.

VAS scores before and after SCN blocks in patients with vertebral fractures were higher than those in patients without vertebral fractures, but did not differ significantly.

### Clinical manifestation before surgery

Nineteen subjects (11 females and 8 males; average age 64.9 years; age range: 41–86 years) required surgical release of the SCN because of intractable symptoms (Table 4). Of these 19 subjects, 11 had a past history of lumbar spine surgery. Of these 11 subjects, 8 reported no improvement in any symptoms after spine surgery, suggesting that those surgeries were perhaps unnecessary. Five cases (cases 1, 4, 10, 12, and 14) had previous vertebral fractures at the thoraco-lumbar junction or upper lumbar region. Two patients (cases 3 and 11) had thoracic outlet syndrome. Otherwise, there were no neurological disorders unrelated to SCN symptoms.

**Table 4:** **Summary of the 19 cases that underwent surgical SCN release**

**Case no.**	**Age**	**Sex**	**Previous spine surgeries**	**Gait**	**Leg symptoms** ^**b**^	**Limitation of lumbar motion**	**Disease duration**	**Pre-op VAS**	**Pre-op RDQ**
1	71	F	Foraminotomy^a^		Tingling^b^: R buttock to calf	F^#^E	3 yr	80	19
2	60	M	Multiple spine surgeries^a^	HW	Pain^b^: R lateral thigh to below knee	F^#^E	12 yr	79	17
3	41	M		PL	Tingling^b^: B thigh to foot sole	F^#^E	6 mon	80	19
4	83	F		PL	Pain: L buttock to lateral thigh	E	5 yr	80	17
5	56	M				F^#^	6 mon	70	9
6	78	F	Lumbosacral fusion		Tingling: L lateral buttock to groin	F	30 yr	65	13
7	61	M		PL	Pain^b^: L lateral calf	F^#^	6 mon	35	13
8	48	F	Herniotomy^a^		Tingling: B buttock to calf	F^#^E	1 yr	100	18
9	56	M	Lumbosacral fusion		Pain: R buttock to calf	F^#^E	1 mon	75	21
10	70	F	Lumbosacral fusion^a^	PL	Pain^b^: L lateral thigh to calf	FE	15 yr	85	9
11	57	F		PL	Pain^b^: B lateral thigh to calf	FE	6 yr	80	20
12	84	M	Laminectomy^a^	PL		FE	1 yr	75	21
13	68	F	Lumbosacral fusion	UW	Tingling: B anterior thigh to toes	FE	30 yr	80	21
14	86	F	Lumbosacral fusion^a^		Pain^b^: B lateral thigh to calf	F	4 yr	100	15
15	75	F	Lumbar fusion^a^		Tingling^a^: R anterolateral thigh and pain: R toes		9 yr	60	12
16	78	F		PL	Pain: R buttock to lateral foot	F^#^E	3 yr	80	16
17	51	M			Pain^b^: R groin and anterior thigh	F^#^	4 yr	50	7
18	44	M				F^#^	10 yr	50	12
19	67	F	Twice laminectomies^a^		Pain^b^: L calf to lateral foot		10 yr	75	5

*SCN* superior cluneal nerve, *no.* number, *op* operation, *VAS* visual analog scale (maximum 100 mm), *RDQ* Roland-Morris Disability Questionnaire, *f* female, *m* male, *LBP* low back pain, *R* right, *L* left, *B* bilateral, *HW* hardly able to walk, *PL* painful limping, *UW* unable to walk, *FE* flexion and extension, *F* flexion, *E* extension, *yr* year(s), *mon* month(s), *F* ^*#*^ coupling of rotation to the side contralateral to symptoms aggravated further than flexion of the affected side alone.

^a^Unnecessary spine surgeries.

^b^Degree of leg symptoms was higher than the degree of LBP.

**Figure 6:**
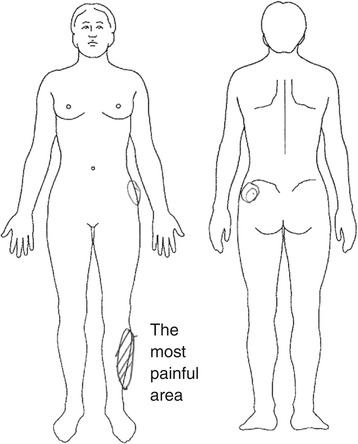
**Clinical manifestations in case 7.** Image drawn by the patient showing leg pain remote from the iliac crest.

Sixteen subjects (84%) had leg pain or tingling. Of these 16 subjects, 10 reported that the leg symptom was more severe than the LBP. Fourteen subjects had leg symptoms spreading from the iliac crest buttock area and the remaining two had leg symptoms remote from the iliac crest (cases 7 and 19) (Figure 6). From gait analysis, 9 of these subjects showed painful limping (Additional file 1 and Additional file 2). Two subjects (cases 2 and 13) could hardly walk because of leg pain. These patients consistently reported that they were unable to walk when we asked them to tighten their buttocks during walking.

**Figure 7:**
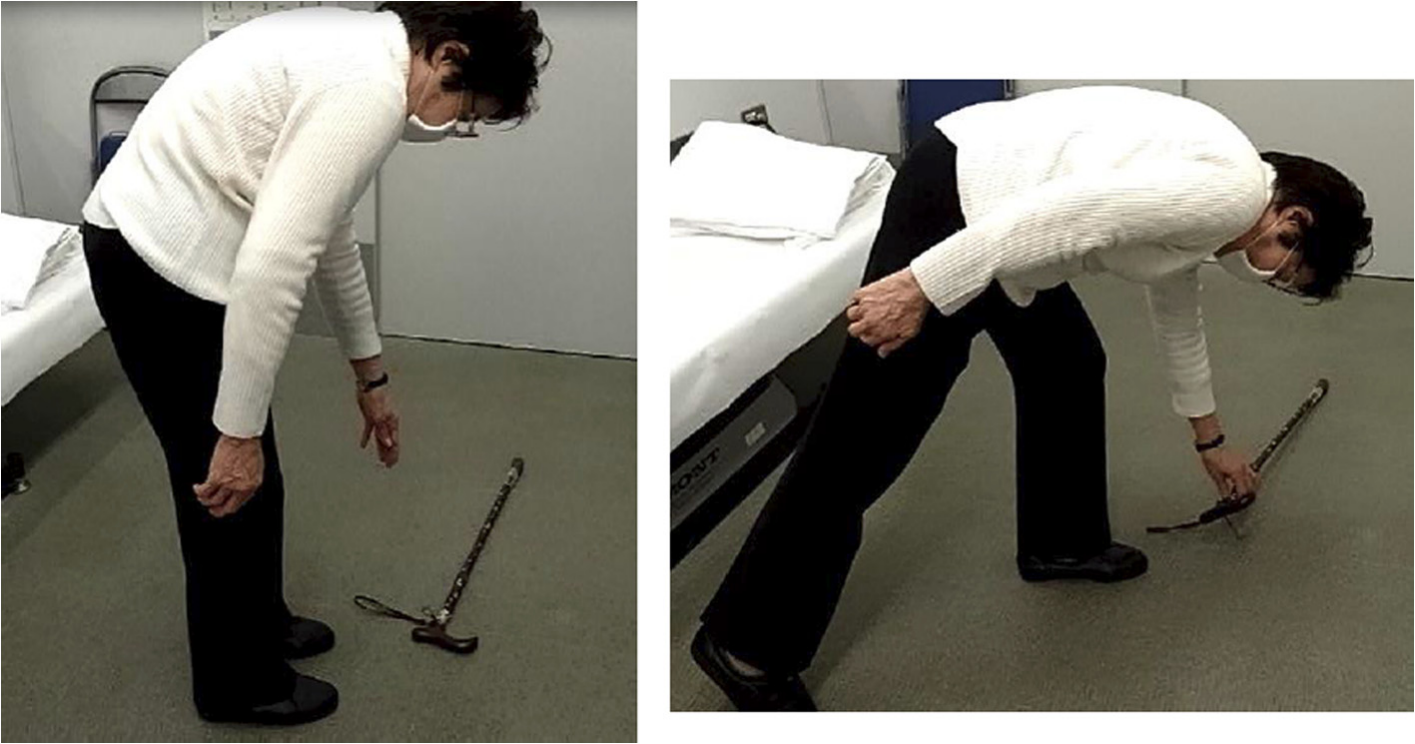
**Pseudo limitation in lumbar flexion in case 16.** Remarkable limitation in flexion had been very disruptive to her daily living and she is showing that she had been unable to pick up her stick from the floor **(A)**. But, she could pick it up when the right hip was extended **(B)**.

Of the 19 subjects, limitation of lumbar motion was noted in flexion (*n* = 6), extension (*n* = 1), or both (*n* = 10). Of the 16 cases with limitation in flexion, 10 reported that coupling of rotation or lateral bending to the side contralateral to their symptoms further aggravated their symptoms (Additional file 3 and Additional file 4). Conversely, three subjects (cases 1, 16, and 17) reported that extension of the ipsilateral hip reduced pain during flexion (Figure 7). Ten patients (cases 1–5, 8–10, 16, and 17) reported that pushing just above the iliac crest by their own hand reduced the symptoms during painful motion, such as flexion/extension and walking (see “Case report 3” section and Additional file 1).

### Surgical observations

In all 19 subjects requiring SCN release, at least two branches of the SCN were found passing underneath a mixture of superficial layers of thoraco-lumbar fascia and the gluteal fascia where it tightly attaches to the iliac crest (Figure 1). Unexpectedly, severe SCN constriction within a bony groove on the iliac crest was never found. During SCN release, anastomosing and bifurcating branches were consistently entrapped underneath the fascia with adhesion to the fascia or surrounding soft tissue and were often pale, suggesting ischemia.

### Surgical outcome

Surgical outcomes are summarized in Table 5. There were no intraoperative surgery-related complications. Average VAS and RDQ scores, indicating significant improvement (*p* < 0.05), decreased from 74 to 35 and from 15.0 to 7.4, respectively. All subjects who had limitation of lumbar motion before surgery reported improvement.

**Table 5:** **Summary of surgical outcomes**

**Case**	**No. of inj.**	**Duration of effect of inj.**	**Post-op relief in leg symptoms**	**Remaining symptoms and remarks**	**VAS (mm) at follow-up**	**RDQ at follow-up**	**Follow-up periods (mon)**
1	90	3 d	Complete		17	1	47
2	33	3 d	Relief for 26 mon	Thigh pain recurred 26 mon after surgery	70	9	37
3	25	3.5 d	Almost complete	Subtle discomfort in R sole	0	0	30
4	37	1.5 d	Relief for 7 mon	Buttock pain recurred 7 mon after surgery	50	11	20
5	9	3 d		Occasional LBP	40	8	23
6	5	3 d	No improvement	50% relief only for LBP	29	9	8
7	4	0.5 d	Complete		5	2	12
8	5	3 hr	70% relief	Calf pain and limitation in lumbar extension remained	25	9	6
9	3	3 d	Complete		0	0	12
10	11	1 d	70% relief for 3 mon	Leg tingling recurred 3 mon after surgery	70	9	7
11	23	1 d	50% relief for 8 mon	LBP recurred 8 mon after surgery	50	13	8
12	3	1.5 d			0	0	14
13	9	2 hr	No improvement	Temporal relief only for LBP; LBP recurred 1 mon after surgery	40	16	13
14	4	1 d	Relief for 3 weeks	Leg pain recurred 3 weeks after surgery	100	16	12
15	4	2.5 hr	Thigh tingling completely relieved	Toes pain was unchanged	0	14	8
16	4	1 hr	60% relief in calf to foot pain	Posterior thigh pain unchanged	30	9	8
17	3	10 d	Almost complete	Occasional groin pain	10	0	5
18	3	0.5 d		Occasional LBP	17	9	14
19	7	2.5 hr	No improvement	Lateral foot pain unchanged but gradually aggravated during the follow-up period	80	7	6

*LBP* low back pain, *inj.* injection, *mon* month(s), *d* day(s), *hr* hour(s).

In the 16 subjects with leg symptoms, surgery resulted in complete relief in 3 (cases 1, 7, and 9), almost complete relief in 2 (cases 3 and 17), no improvement in 3 (cases 6, 13, and 19), and partial improvement in the remaining 8 cases at least at the 6 months follow-up. Five subjects (cases 2, 4, 10, 11, and 14) had temporal pain relief following surgery, but had recurrence of symptoms within an average of 9.4 months (range: 3–26).

Of the 16 subjects who had leg symptoms, subjects with shorter duration of symptoms (3 years or less) had a significantly higher ratio of excellent outcomes (complete or almost complete relief) than the remaining subjects with longer duration (more than 3 years) (4/6 vs 1/10, *p* < 0.05). Patients with more than 3 days of effectiveness from SCN blocks had a significantly higher ratio of excellent outcome than the patients with less than 3 days relief (4/6 vs 1/10, *p* < 0.05).

### Surgical case reports

#### Case 1

A 67-year-old woman reported a 3-year history of disabling right leg tingling superimposed on LBP for the preceding several years. She had a 10-year history of rheumatoid arthritis and was treated with 6 mg/week methotrexate. At age 65, she had delayed paraplegia after a collapsed vertebral fracture at T12. Urinary incontinence and weakness below the psoas muscles were successfully treated by posterior thoraco-lumbar surgery combined with pedicle subtraction osteotomy at T12. After surgery, however, the right leg tingling was unchanged. She also complained that the range of motion of the lumbar spine was limited in all directions. In particular, flexion was highly limited and forced flexion reproduced leg tingling radiating to the calf. Her finger-to-floor distance was 60 cm. Selective L5 nerve root infiltrations were repeated and resulted in temporal improvement of leg tingling to some degree. With a tentative diagnosis of L5/S1 foraminal stenosis, a foraminotomy was attempted at age of 67, with no resulting change in symptoms. After the lumber surgery, we observed a point of tenderness to palpation slightly below the iliac crest (7 cm laterally to the midline, 1 cm inferiorly to the iliac crest). During palpation, she reported recurrence of the leg tingling in the calf region. From age 68, the SCN block was repeated 90 times. Every time, she reported reappearance of leg tingling during the SCN block and, soon after, clear improvement in leg tingling that continued for 3 days. Although oral prescription of opioids, lumbar sympathetic nerve blocks, epidural blocks, and facet blocks were also attempted, she reported that SCN blocks worked much better than these. At age 71, she was listed as a candidate for spinal cord stimulator therapy in the Department of Anesthesiology. At this time, we decided that an attempt to surgically release the SCN was indicated.

During surgery, three branches were identified passing beneath the thoraco-lumbar and gluteal fascia over the iliac crest. The branches were compressed by the fascia with adhesions and released by opening the fascia. She reported disappearance of leg tingling when she awoke from the general anesthesia. Leg tingling and limitation in lumbar motion have not recurred at 47 months after surgery.

#### Case 2

A 58-year-old man presented with complaints of severe pain in his right lower leg, which had gradually developed over 10 years. Repetitive spine and knee surgeries were performed for this leg pain, including L4 nerve root decompression at age 48, medial meniscectomy at age 51, L4/5 foraminotomy at age 52, L4/5 transforaminal intervertebral body fusion (TLIF) at age 52, and L5/S1 TLIF at age 58, none of which produce pain relief. The pain was so severe that he often refused to walk. A neurologic examination revealed that the right patellar tendon reflex and the bilateral Achilles tendon reflexes were reduced. No sensory disturbance was apparent even in the buttock area, and bowel and bladder functions were normal. He had point tenderness over the left iliac crest 8 cm from the midline and pain radiating to the posterolateral thigh from this location. SCN blocks were repeated 33 times with consistent but temporary pain relief. SCN surgery was done at age of 60. He noticed disappearance of thigh pain when awakening from the general anesthesia. Two years passed uneventfully, but thigh pain gradually recurred 26 months after surgery.

#### Case 3

A 41-year-old man presented complaining of bilateral leg tingling with tightness predominantly on the right, which had developed gradually over 1 month following acute onset of LBP. He had a history of posttraumatic thoracic outlet syndrome causing left arm tingling at age 32. Because the arm tingling was not fully controlled by spinal cord stimulation performed at age 34, a first rib surgical resection was done at age 43, resulting in almost complete elimination of the arm symptoms.

At first examination, he could walk, but it seemed very painful. Forward and backward bending of the lumbar spine was highly limited, especially forward bending. Pushing on his back proximal to the iliac crests using his hands reduced pain during painful motion (Additional file 1). A lower limb neurological examination of both motor and sensory was unremarkable. He had tenderness at the SCN tender point on both sides. Palpation on the tender point consistently induced LBP and leg tingling radiating from the buttocks to the soles of his feet on both sides. Bilateral SCN blocks consistently resulted in a 50% improvement in leg tingling. He also reported ED after every injection, which continued during for 3.5 days until the effect of SCN blocks disappeared. In spite of the temporary ED, he repeatedly requested SCN blocks because of intractable tingling. Consequently, SCN blocks were repeated 25 times over 5 months without substantial permanent change in painful walking.

At 41 years of age, SCN release was performed bilaterally. Two days after surgery, he reported disappearance of leg tingling. At 4 days after surgery, he could walk smoothly without any symptoms (Additional file 2). Range of motion of the lumbar spine was completely pain free. After 30 months, no pain or symptoms reappeared, although he reported that a very subtle discomfort remained in sole of his right foot.

## Discussion

SCN disorder patients were significantly older and had significantly more vertebral fractures in the thoraco-lumbar or lumbar spine than the remaining patients suffering from LBP and/or leg symptoms enrolled in this study. It should be noted that approximately 20% of patients with vertebral fractures at the thoraco-lumbar junction may be treatable by SCN blocks, as shown in the illustrative case report. It is suggested that vertebral fractures may elicit preexisting, but asymptomatic or subclinical, SCN entrapment over the iliac crest by irritation of the SCN at its origin from unstable facet joints and/or stretching of the SCN due to increased kyphosis of the spine.

From 1930 to 1960, cluneal nerve syndrome was sporadically reported as a cause of LBP [4,24-27]. Strong and Davila used diagnostic criteria for SCN disorder similar to ours [4]. Their diagnostic criteria were that (i) a constant tender point no larger than 2 cm in diameter was situated in the low lumbar or episacral area and (ii) an injection with 2 ml of 1% procaine eliminated the trigger point and LBP; preferably, relief should have been obtained by injection at least twice [4]. However, their criteria lacked a clear definition of pain relief and did not limit the tender point to the iliac crest where the medial branch of the SCN passed through an osteofibrous tunnel. Strong and Davila reported that 9.8% of their patients (*N* = 122) who were admitted to the hospital because of LBP had SCN disorder [4].

In the current study, the diagnosis of SCN disorder was made solely based on two clinical criteria: 1) maximal tenderness existing over the iliac crest, even when tenderness also exists elsewhere, for example, at the thoraco-lumbar junction, and 2) the chief complaint being reproduced by palpation of the iliac crest. In our study, 113 (14%) of the 834 patients who registered were diagnosed with suspected SCN disorder. Ninety-one (11%) of these subjects experienced more than a 20 mm decrease in VAS scores after the first nerve block injection and 70 subjects (8%) experienced more than a 50% decrease in VAS scores after the injection. Patients who previously had bone harvested from the posterior iliac crest were not included in our study. It is likely that SCN disorder is not a rare clinical entity and is more commonly the result of spontaneous entrapment of the nerve than a nerve injury during bone harvest, as Trescot stated [18].

Trescot mentioned that entrapment of the SCN caused referred pain down the leg, potentially all the way to the foot, and that this “pseudo-sciatica” would clinically mimic radiculopathy due to lumbar disc herniation or lumbar spinal canal stenosis [18]. Strong and Davila surgically treated 30 patients by deafferentation of SCN [4]. Although the chief complaints were limited only to LBP, 53.8% had referred leg pain in all areas of the leg. In the present study, approximately 50% of the SCN disorder patients had leg symptoms. This ratio was higher (89%) in 19 cases that required surgeries because of severe symptoms. It should be noted that 8 out of these 19 cases had a history of possibly unnecessary lumbar surgeries.

Clinical manifestations of leg symptoms are quite variable, especially in area of symptoms and aggravating posture. Strong and Davila described that localization of pain was difficult for the patient [4]. In their cases, patients complained of leg symptoms in a variety of areas from groin to sole on the foot. In the majority, pain radiated from the iliac crest down to the leg, but a few had pain remote from the iliac crest. Previous anatomical studies did not explain why SCN causes “pseudo-sciatica” because the SCN was thought to be composed of sensory branches of the dorsal rami of T11-L4 and to travel over the iliac crest to supply the skin overlying the posteromedial area of the buttock [1-3]. To resolve this issue, Konno et al. recently performed an anatomical study using six cadavers to identify the level of origin of the nerve passing through the osteofibrous tunnel [28]. Of the ten specimens that which had the medial branch of the SCN passing through the tunnel, the nerve could be traced medially to the L3 in one, L4 in five, and L5 foramina in four. In addition, the most superior branch of the medial cluneal nerve (MCN) often anastomoses with medial branches of the SCN distally, inferior to the iliac crest in the subcutaneous tissue [29,30]. MCNs are composed of sensory branches of the dorsal rami of S1 to S3 [29,30]. The broader origin of the SCNs and the evidence that predominantly the L4 and L5 lateral branches pass through the tunnel and thereafter anastomose with the S1 and S2 lateral branches may explain why SCN disorder could cause leg symptoms in variable areas and mimic sciatica.

It is likely that the limitation of lumbar motion and leg symptoms could lead to misdiagnosis and unnecessary spine surgeries. Patients with severe symptoms often present characteristic signs suggesting irritable friction of the SCN under the fascia. Characteristic painful limping and limitation in lumbar motion differ from those seen in spinal disorders. Tightening buttocks often aggravates pain during gait, suggesting that constriction of the gluteus muscles squeezes the SCN at the fascial orifice. Patients often realized that pushing above the iliac crest with their hands reduced symptoms. This suggests that immobilizing the proximal portion of the SCN inhibits a continuous piston-like movement of the SCN under the fascia.

Symptoms were often aggravated during flexion. Coupling rotation to contralateral side and flexion further aggravated symptoms. Flexion and contralateral rotation would strain the SCN. Conversely, extension of ipsilateral hip reduced pain during flexion. This may be because hip extension loosens the SCN. On careful observation, this is seen to be a pseudo-limitation of lumbar flexion. On the other hand, SCN patients often reported that extension of the lumbar spine aggravated the symptoms. This is similar to Phalen’s and reverse Phalen’s maneuvers used as screening methods for carpal tunnel syndrome. These characteristic signs are useful as a provocative examination maneuver to screen and differentiate SCN disorders from lumbar disorders. Patients who have true sciatica due to spinal canal or foraminal lesion usually have tenderness in the gluteal regions (Valleix’s points). Although they may also have tenderness on the iliac crest, palpation of this point would not reproduce the leg symptoms.

Spine surgeons should be aware that SCN disorder is not rare and may cause not only LBP but also leg symptoms. The key to diagnosing SCN disorder is palpation of the tender point and determining if this palpation provokes the symptoms even when symptoms are remote from iliac crest. As stated by Strong and Davila, the patient is rarely aware of the presence of a trigger area in the back [4]. SCN blocks are useful not only for obtaining pain relief but also to confirm the diagnosis by pain relief just after injection. Patients may report provocation of leg symptoms during injection. We cannot explain the underlying mechanism of ED as a rare complication of SCN blocks. Even knowing this complication, we still consider SCN blocks to be mandatory for accurate diagnosis of this clinical entity and control of intractable symptoms.

The term “osteofibrous tunnel” implies severe constriction in the bony groove as illustrated by Kuniya et al. [10]. In spite of that, surgery in our study cohort was performed only for selected patients with severe symptoms, the Ilium never being directly involved in entrapment. We recently performed SCN surgery in an additional 26 cases not included in this study. Of these, two patients had SCN constriction under the fascia tightly attached over the bony groove on the ilium. It is likely that true constriction in the “osteofibrous tunnel” may be extremely rare and repetitive friction of the SCN under the fascia even without direct compression by the ilium could cause severe symptoms. Our results indicated that patients with a shorter duration of symptoms and longer duration of pain relief after SCN injection are good candidate for surgery. Selecting these patients as surgical candidate would improve over surgical outcome.

## Conclusions

Patients with SCN disorders comprised about 10% of all patients presenting with a chief complaint of LBP and/or leg symptoms; thus, this is not a rare clinical entity. Approximately 50% of SCN disorder patients had leg symptoms. Eighty-five percent of SCN disorder patients experienced clinical pain relief from repeated nerve block injections, up to three times, when clinical pain relief was defined as more than a 20 mm decrease in VAS. When patients demonstrated a localized tender point on the iliac crest and palpation of this point reproduced their chief complaints, a SCN block proved to be an effective treatment modality.

## Additional files

Additional file 1: Video 1. Painful limping in case 3. Pushing his back proximal to iliac crests by his hands reduced pain during painful motion. Additional file 2: Video 2. Video taken 4 days after surgery in case 3. Note complete recovery in limping was obtained. Additional file 3: Video 3. Limitation in flexion in case 9. Sharp leg pain and tingling on the right spreading from iliac crest reappeared during flexion. He reported that coupling of flexion and left rotation consistently reproduced leg pain. He also reported that pushing just above the right iliac crest by his own hand diminished the symptoms during painful motion. Additional file 4: Video 4. No limitation in flexion after surgery in case 9. One week after surgery shows full range of flexion obtained with no pain reappearance.

## Abbreviations

SCN: Superior cluneal nerve
LBP: Low back pain
VAS: Visual analog scale
RDQ: Roland-Morris Disability Questionnaire
ED: Erectile dysfunction
